# Movement Variability and Perceived Motor Competence in Children with High or Low Risk Willingness in a Virtual Playground

**DOI:** 10.3390/children12060796

**Published:** 2025-06-18

**Authors:** Lise Storli, Håvard Lorås

**Affiliations:** 1 Department of Physical Education and Health, Queen Maud University College of Early Childhood Education, 7044 Trondheim, Norway; havard.loras@ntnu.no; 2 Department of Teacher Education, Faculty of Social and Educational Sciences, Norwegian University of Science and Technology (NTNU), 7012 Trondheim, Norway

**Keywords:** risk management, risky play, motor development, motor control, locomotor, balance

## Abstract

Background: The current study explores the relationship between children’s risk willingness and their motor behavior in a virtual playground setting and its association with perceived gross motor competence. Methods: A total of 96 children aged seven to ten participated. They were categorized into high-risk-willingness (HRW) and low-risk-willingness (LRW) groups based on their exploratory behavior and engagement with riskier zones and tasks in the playground. Using whole-body motion capture and virtual reality data, the children’s motor behavior and variability were analyzed alongside self-reported perceived gross motor competence. Results: The results indicated that HRW children displayed significantly greater movement variability, including higher joint movement variability and increased exploration of challenging areas compared to LRW children. HRW children also covered greater distances, moved faster, and exhibited more frequent acceleration changes. Conclusions: These findings suggest that higher risk willingness is associated with greater adaptability and flexibility in motor behavior, aligned with the concept of freeing degrees of freedom. In contrast, no significant differences were found in perceived gross motor competence ratings between HRW and LRW groups. This indicates that perceived motor competence may not directly influence children’s willingness to take risks or their motor behavior in exploratory play. These findings emphasize the importance of studying dynamic interactions between risk-taking, motor behavior, and self-perception to understand the development of adaptive motor skills through risky play.

## 1. Introduction

Children’s risk willingness as a psychological construct involves emotional and physical processes and can be defined as a child’s propensity for taking risks [1]. Risk willingness influences the degree to which a child will engage in variations of risky play, a form of play that allows children to test their physical and cognitive limits, explore boundaries, and engage in activities with a potential for getting injured [2,3]. Risky play offers children physical and emotional experiences [4] and provides opportunities for exhilarating positive emotions and confronting fears [5]. Furthermore, risky play has been shown to physically promote higher levels of physical activity and enhance children’s ability to manage precarious situations [6]. Psychologically, it fosters resilience, problem-solving abilities, and conflict-resolution skills; socially, it supports the development of cooperation, communication, and a sense of belonging [5,7,8]. Thus, risky play is considered vital for children’s health and development [9]. Many children are naturally inclined toward risky play, often describing sensations such as a “tickling in the stomach” or “getting butterflies” during these activities [3]. However, individual differences in children’s risky play exist, and, to date, previous research has focused primarily on environmental and task-related constraints influencing aspects of children’s risky play [10,11,12]. Individual constraints, such as a child’s perception of fear, can also influence their willingness to engage in risk-taking behaviors during play, thus making risk willingness important to understand as a potential prerequisite for risky play.

Children’s risk willingness can be linked to personality traits such as sensation-seeking, which describes a child’s inclination toward varied, complex, and intense sensations and experiences [13]. Children with higher thrill-seeking tendencies have been found to engage in more risky play, which can help them develop risk management skills [14,15]. Other personal factors that have been found to affect risk willingness are the child’s age and gender. Older children are often more willing to take risks, and boys tend to take more risks than girls [16]. A child’s previous positive and/or negative experiences with risks might also show differences in risk willingness [4]. While personality traits might affect a child’s risk willingness, other factors, such as parental influence, can also have an impact. Overprotective or risk-averse parents can lead to low risk willingness in children [17,18].

According to dynamic risk management theory [14], children’s risk willingness has a reciprocal relationship with their risk handling. The latter term refers to the execution of actions the child takes and, according to the theory, manifests in the child’s motor behavior. Risk handling is thus predominantly considered to be a motor process involving emergent and observable movements when engaging in risky tasks. These actions allow children to handle certain task-specific physical risks, depending upon the individual’s motor development of fundamental movement systems, such as locomotion, balance, and object handling [19], while at the same time influencing the dynamic process of motor development. As a logical entailment of the proposed reciprocal and dynamic relationship between children’s risk willingness and risk handling, it could be hypothesized that observable actions (i.e., risk handling) are different between children displaying high risk willingness compared to children displaying low risk willingness in play.

There seems to be limited knowledge about what might constitute potential differences in movements between children with different degrees of risk willingness when they engage in risky tasks. From a dynamical system perspective on motor development, differences and variability in movements and their outcomes are considered key elements [20,21]. Children’s execution of different motor acts is characterized by quantitative and qualitative characteristics of the coordination of their whole-body movements [22], and the variability of this dynamic process, in turn, depends upon how children handle and find individual solutions to the many degrees of freedom present in their bodies [23,24]. This dynamic process of handling degrees of freedom has been conceptualized through the notion of freezing or freeing degrees of freedom, which, according to Bernstein (1967) [25], involves controlling movements or “freezing” through a strategy of reducing the many possible movements to the minimum required. Alternatively, the degrees of freedom can be released gradually, a strategy depicted as “freeing degrees of freedom”, and this involves more variability in movements.

The first strategy of freezing movements can be indicative of simplifying control and less variability in the relationship between the movements displayed, the risky task that is explored, and the intentions to take risks. The latter strategy, alternatively, signifies children exploring a higher number of degrees of freedom combinations and displaying more movement variability in a risky task. A more adaptable and flexible motor repertoire might be associated with freeing degrees of freedom [26,27,28], i.e., more variability in movement during play. In contrast, freezing degrees of freedom (reducing variability) might indicate a less adaptable and flexible motor repertoire [29,30,31]. Children who can use multiple movement strategies might have more opportunities to choose different strategies and be more flexible in their risk-taking behavior, allowing them to manage risks better and potentially be encouraged to explore challenging tasks and be more risk-willing [32,33]. A less flexible and adaptive motor repertoire, on the other hand, might signify fewer possibilities for exploration and risk handling and thus might be associated with reduced risk willingness in children.

A potential psychological factor that can have an influence on both children’s risk willingness and risk handling (motor behavior) is their perceived gross motor competence (PMC). PMC is considered an important factor in motor development that influences emotions, motivation, and behavior [34,35], and the term refers to children’s confidence in their ability to perform various motor skills. These are typically divided into three interrelated skill categories that constitute a child’s gross motor skills: locomotion, object control, and balance/stability [36]. PMC is considered fundamental to participation in physical activities and sport in school and leisure time [36,37,38]. It is, however, not necessarily aligned with children’s actual (measured) gross motor competence [39,40,41]. The research literature has indicated that children can both overestimate and underestimate their motor competence [42,43,44]. Children with high perceived motor competence might trust their ability to adapt and find solutions to motor challenges and exhibit a greater ability to manage degrees of freedom. Thus, they might be inclined to explore risk-taking to test their physical abilities, rooted in a belief that they can handle challenging physical activities [45]. Conversely, children with lower perceived motor competence might avoid risks due to a lack of confidence in their abilities and stick to less risky environments where they can execute safer and less challenging motor tasks. Thus, PMC might influence children’s propensity for taking risks (risk willingness), as well as the execution of actions in risky tasks (risk handling).

### The Current Study

**Figure 1:**
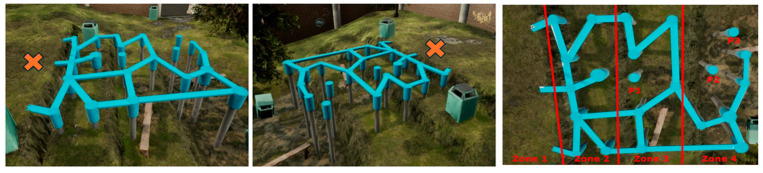
Overview of the virtual reality playground. The image to the right illustrates the four zones and pillars (P1, P2, and P3).

Based on the considerations presented, the objective of the current study was to compare children with high risk willingness to those with low risk willingness on risk handling (movement variability) and aspects of self-rated perceived gross motor competence. Children’s risk willingness and risk handling were examined when they freely explored a custom-made balance beam playground situated in a novel and safe three-dimensional (multimodal) virtual reality city environment (see Figure 1). The seven- to ten-year-old children were allowed to explore the playground without any specific instructions other than “Don’t fall off”. Thus, they were allowed to take risks by moving into pillars and bars at greater heights depending on their own choice, while at the same time, they were allowed to freely move around and explore their individual degrees of freedom while they handled the potential risks. 

The X marks the initial position of the children. The following two research questions are presented:

RQ 1: Do children with high risk willingness in a virtual playground display different motor behavior compared to those with low risk willingness?

RQ 2: Do children with higher risk willingness rate their perceived gross motor competence higher compared to those with low risk willingness in a virtual playground?

## 2. Materials and Methods

This study is part of a larger study that involved several virtual reality scenarios that were further elaborated on in a separate protocol paper [46]. The current study focuses on the last scenario, the playground, due to its unique characteristics of free exploration without any goal-directed task, allowing children’s risk willingness and handling to emerge in a risky play context.

For the overall project, 417 children were recruited to participate. Four schools were invited and agreed to participate in the research. Informed consent was obtained from the children’s guardians before participating, and the children provided their assent before participating in any virtual reality task. Out of these, data from 358 children, ranging from ages seven to ten years old (8.7 ± 0.8 years), constituted a subsample with complete motion capture data and provided ratings of their perceived motor competence, making them eligible for the current study. The sample was equally distributed between the sexes, with 176 girls and 182 boys. The Norwegian Agency for Shared Services in Education and Research (SIKT) ethics board approved the ViRMa project (No. 324155).

### 2.1. Children with High Risk Willingness and Low Risk Willingness

Two groups of children with high risk willingness and low risk willingness, respectively, were identified from the larger subsample based on their behavior in the playground (see Figure 1). The first subgroup (n = 39) was designated as low risk willingness based upon the following criteria: (1) they did not jump on any of the playground pillars in either zone, and (2) they spent most of their time exploring Zones 1–3, i.e., at least one standard deviation below the mean time in Zone 4 for the total sample. Consequently, children were categorized as having high risk willingness (n = 81) if they spent above the mean time exploring in Zone 4 and visited all three pillars at least once.

### 2.2. Whole-Body Motion Capture

**Figure 2:**
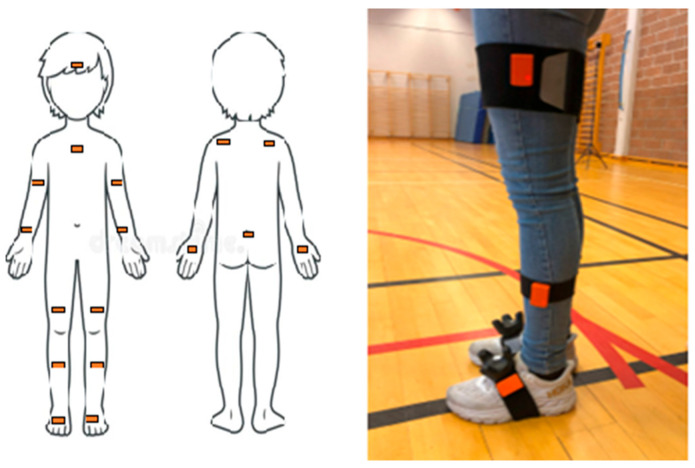
Illustration of the anatomical positioning of the IMUs. The head sensor was positioned on top of the headset. The image to the left illustrates the placement of the lower-body sensors attached with Velcro straps on the lateral side of the body.

This study applied the Xsens motion capture system to designate children’s movements in the virtual playground. The inertial system captured kinematic data from joint rotations and segment movements in three orthogonal directions. Data was sampled at 60 Hz through a proprietary radio protocol at 2.4 GHz [47]. The system comprises 17 wireless sensors (IMUs) and ensures accurate time synchronization across the wireless network within 10 μs [47]. The IMUs were positioned at various anatomical locations with Velcro straps or attached to a specially designed T-shirt (see Figure 2). Each IMU has a size of 47 × 30 × 13 mm and weighs 16 g. The IMU system has been recommended for use in biomechanics, sports, and rehabilitation, as it is highly portable, enables full-body movement data, and is a reliable tool for capturing movement data compared to wired inertial systems [48,49,50].

### 2.3. Virtual Reality Technology

The virtual reality (VR) scenarios were created by a VR company (Nordic Neurotech AS) and utilized HTC VIVE PRO goggles with five HTC VIVE trackers. Two of the trackers were placed on the participants’ feet, enabling them to see transparent feet in the VR environment. The participants did not see any other parts of their body during the task. The test area was defined by positioning four HTC VIVE base stations, creating an area of 5 × 6 m within which the participants could move. There were no other elements in the VR area, only a flat floor. The researchers could always see what the participants looked at in the VR goggles on a separate computer, and data was sampled at 90 Hz.

### 2.4. Virtual Reality Playground

The playground offered play opportunities that required locomotor and stability skills [51]. It was composed of several balance beams that were stuck together and pillars placed around for the participants to jump on (see Figure 1). The playground is situated in a city environment with sounds from passing traffic in the background, as well as sounds from birds, wind, etc., to facilitate the VR immersion experience. The balance beams measured 20 cm in width, and the smallest pillar had a diameter of 30 cm. The various playground zones had the following height above ground: Zone 1 = 0 m; Zone 2 = 0.8 m; Zone 3 = 1.45 m; and Zone 4 = 2.35 m. The heights of the pillars were Pillar 1 at 1.45 m and Pillars 2 and 3 at 2.34 m. If the participant were to fall off the beams or a pillar, the VR screen would turn black, and the child would receive a notification from a prerecorded voice that they had fallen off before they started in Zone 1 again. The participants had three minutes to move around freely on the playground, and if they had a fall, the time continued, as these tasks required the children to take a jump. Two pillars were also located in the highest area, which was categorized as the riskiest zone.

### 2.5. Perceived Motor Competence

The Pictorial Scale of Perceived Movement Skill Competence (PMSC) was used to measure the children’s perceived motor competence. This scale has been validated as acceptable for assessing perceived motor competence among seven- to ten-year-old Norwegian children [52]. The scale evaluates children’s self-perceived abilities across several domains, including stability skills (e.g., balancing), locomotion skills (e.g., running, galloping, and hopping), object control skills (e.g., bouncing, catching, and throwing), and active play (e.g., biking, rope climbing, and swimming) [53,54]. The object control skills were not included in this study, as none of these were performed during the VR playground tasks. The PMSC uses pictures of a child performing various activities, with two pictures where one is of a child performing the task better than the other. The children rated their performance on a 1 to 4 scale depending on their chosen picture. If they chose the picture of the child performing the task in an incompetent way, they perceived their competence based on the following choices: (1) “not too good” or (2) “sort of good”. In contrast, if they chose the picture illustrating a good performance, they selected between (3) “pretty good” and (4) “really good”. The scale had separate versions for boys and girls.

### 2.6. Procedure

One child at a time was escorted from their classroom to the test location at their respective schools. The virtual reality scenarios took approximately 10–15 min to complete, including the last scenario analyzed in the current study that lasted three minutes. After entering the test location, the virtual reality and motion capture equipment were mounted. All children performed an introductory task to become familiar with the virtual reality environment and to ensure that the participating child did not experience cybersickness. After completing the virtual reality scenarios, the child was escorted to a separate room nearby for an interview that lasted approximately 10–15 min.

### 2.7. Playground Motor Behavior Analysis

The custom-made virtual reality software collected data on the position of each individual child on the playground, relative to the different zones and pillars illustrated in Figure 1, as well as whether the child fell off the virtual playground. These data were exported as a .txt file and processed in MATLAB R2023 (MathWorks Inc., Natick, MA, USA) with in-house algorithms to obtain the following variables analyzed in the current study: the number of visits to each pillar and the time spent in each of the four zones.

For the kinematic measures from Xsens, the Xsens MVN system, version 2022.02 (Xsens, Enschede, The Netherlands) preprocessed all the motion capture files to ensure each file’s transfer and quality. After creating MVNX files, raw data was exported into MATLAB for further analysis with custom-made algorithms. After inspections of frequency spectra using the periodogram method, a low-pass, zero-phase, fourth-order Butterworth filter was applied to filter the movement data. The cutoff values for the filter ranged between 5 and 15 Hz, depending on the specific frequency content of the analyzed movements. In previous studies, short-duration discrete movement phases and joint angular amplitudes have been typically assessed using range of motion (ROM) measurements. However, joint-specific ROM does not adequately reflect the dynamic variability in children’s ability to manage degrees of freedom. Consequently, this study computed the standard deviation (SD) across the entire movement series [12]. The SD is a widely used metric for examining variability in the joint range of motion and other coordination patterns [55,56,57,58]. The children’s handling of degrees of freedom was analyzed from the following whole-body movements: head pitch and yaw, shoulder abduction–adduction, and elbow flexion–extension for the upper body. For lower-body movements, hip flexion–extension, knee flexion–extension, ankle dorsiflexion–extension, and movement of foot segments in mediolateral, anterior–posterior, and vertical directions [59,60,61] were analyzed.

### 2.8. Statistical Analysis

The Shapiro–Wilk tests, inspections of Q-Q plots, and histograms were used to infer the normal distribution of study variables. Before conducting further analyses, we examined intercorrelations between the measures using Pearson’s r. This was carried out to identify any strong correlations (r ≥ 0.70), which would suggest that the variables share substantial variance and may not represent distinct constructs. In the remaining data, close inspection of the correlation matrix indicated strong bilateral correlations for movements of the major joints: ankles, knees, hips, elbows, and shoulders, as well as for the height of the forearm. Thus, the means of right/left movements were used in the remaining analyses.

Movement variability (Research Question 1) and perceived motor competence (Research Question 2) were compared between children with high risk willingness and children with low risk willingness in the virtual playground with independent sample *t*-tests and chi-square tests. For the *t*-tests, Cohen’s d was applied to measure the effect size [62], which was interpreted as small = 0.2; moderate = 0.5; and large = 0.8 [63]. All statistical calculations were performed using Predictive Analytics Software (PASW, IBM, 275 United States; previously SPSS) Version 26.0 with alpha = 0.05 as the criterion for statistical significance.

## 3. Results

Descriptive statistics for demographic variables can be found in Table 1. The high-risk-willingness group of children was significantly older (t(94) = 4.87, *p* < 0.001, d = 1.08) and had a higher average body height (t(94) = 4.07, *p* < 0.001, d = 0.92) compared to the low-risk-willingness group of children. No significant difference was found between groups in the distribution of gender (χ^2^(1, n = 96) = 1.90, *p* = 0.168, V = 0.14) or in previous virtual reality experiences (χ^2^(1, n = 96) = 1.91, *p* = 0.167, V = 0.14). Furthermore, the proportion who experienced some form of virtual reality sickness (χ^2^(1, n = 96) = 0.33, *p* = 0.563, V = 0.06) or wanted to try virtual reality again was not significantly different between groups (χ^2^(1, n = 96) = 0.52, *p* = 0.472, V = 0.07).

### 3.1. Analysis for Research Question 1: Motor Behavior and Risk Willingness in the Playground

Descriptive statistics for motor behavior variables can be found in Table 2. As expected, the high-risk-willingness group visited more pillars (t(94) = 13.48, *p* < 0.001, ΔM = 5.85 [95% CI: 4.99, 6.71], d = 2.00 [95% CI: 2.38, 3.60]) and spent less time exploring Zone 1 (t(94) = 6.20, *p* < 0.001, ΔM = 10.0% [95% CI: 6, 13], d = 1.38 [95% CI: 0.90, 1.85]) and Zone 2 (t(94) = 6.20, *p* < 0.001, ΔM = 20.0% [95% CI: 0.17, 0.24], d = 2.53 [95% CI: 1.96, 3.09]) in the playground. Consequently, no significant difference was found in time spent in Zone 3 (t(94) = 1.07, *p* = 0.29, ΔM = 0.02% [95% CI: −0.06, 0.02], d = 0.24 [95% CI: −0.67, 0.20]), whereas the high-risk-willingness children spent considerably more time exploring Zone 4 compared to the low-risk-willingness group of children (t(94) = 20.22, *p* < 0.001, ΔM = 28% [95% CI: 0.25, 0.30], d = 4.50 [95% CI: 3.72, 5.27]). Further independent *t*-tests for spatiotemporal variables measured in the playground indicated that the high-risk-willingness group moved a longer total distance during the three-minute period (t(94) = 5.76, *p* < 0.001, ΔM = 15.40 [95% CI: 10.09, 20.72], d = 1.34 [95% CI: 0.84, 1.84]) and with higher average velocity (t(94) = 5.92, *p* < 0.001, ΔM = 0.11 [95% CI: 0.07, 0.15], d = 1.32 [95% CI: 0.83, 1.79]) compared to the low-risk-willingness group. The mean acceleration was also higher in the children with higher risk willingness (t(94) = 6.36, *p* < 0.001, ΔM = 0.17 [95% CI = 0.11, 0.22], d = 1.48 [95% CI: 0.97, 1.99]). There was no significant difference in the proportion of children falling off the virtual playground (χ^2^(1, n = 96) = 0.05, *p* = 0.82, V = 0.02).

Between-group comparisons with independent sample *t*-tests for whole-body movement variability indicated no difference in the variability of head movements: pitch (t(94) = 0.87, *p* = 0.39, ΔM = 1.61 [95% CI: −5.32, 2.09], d = 0.20 [95% CI: −0.66, 0.26]) and yaw (t(94) = 1.47, *p* = 0.14, ΔM = 1.84 [95% CI: [−4.33, 0.64], d = 0.34 [95% CI: −0.80, 0.12]). For other upper body movements, movements of the shoulder joints (t(94) = 4.68, *p* < 0.001, ΔM = 3.35 [95% CI = 1.93, 4.77], d = 1.09 [95% CI: 0.60, 1.57]) as well as the mean (t(94) = 3.13, *p* = 0.002, ΔM = 0.03 [95% CI = 0.01, 0.05], d = 0.73 [95% CI: 0.26, 1.19]) and variability (t(94) = 4.49, *p* < 0.001, ΔM = 0.03 [95% CI = 0.01, 0.04], d = 1.05 [95% CI: 0.56, 1.53]) of arm lifts were significantly different, with higher variability in the high-risk-willingness group. No significant difference was found between the groups for the variability of elbow movements: t(94) = 1.63, *p* = 0.11, ΔM = 4.07 [95% CI: −9.05, 0.91], d = 0.38 [95% CI: −0.85, 0.08].

Overall, the children with high risk willingness demonstrated significantly higher variability in lower-body movements, including hip (t(94) = 6.29, *p* < 0.001, ΔM = 4.85 [95% CI = 3.32, 6.38], d = 1.47 [95% CI: 0.96, 1.97]), knee (t(94) = 4.56, *p* < 0.001, ΔM = 3.15 [95% CI = 1.77, 4.52], d = 1.06 [95% CI: 0.58, 1.54]), and ankle joints (t(94) = 8.93, *p* < 0.001, ΔM = 3.30 [95% CI = 2.57, 4.04], d = 2.08 [95% CI: 1.53, 2.63]), as well as for the distances between feet in the three orthogonal directions: anterior–posterior (t(94) = 10.07, *p* < 0.001, ΔM = 0.04 [95% CI = 0.03, 0.05], d = 2.35 [95% CI: 1.77, 2.92]), medial–lateral (t(94) = 8.43, *p* < 0.001, ΔM = 0.04 [95% CI = 0.03, 0.05], d = 1.97 [95% CI: 1.42, 2.50]), and vertical (t(94) = 4.48, *p* < 0.001, ΔM = 0.01 [95% CI = 0.004, 0.01], d = 1.04 [95% CI: 0.55, 1.52]).

### 3.2. Analysis for Research Question 2: Perceived Motor Competence and Risk Willingness in Playground

Descriptive statistics for the ratings of perceived motor competence can be found in Table 3. From the items of perceived locomotor skills, only the running item (t(94) = 2.67, *p* = 0.009, ΔM = 0.39 [95% CI = 0.10, 0.67], d = 0.61 [95% CI: 0.15, 1.06]) was significantly different between groups, with higher ratings from the children in the high-risk-willingness group. No other items on perceived locomotor competence were significantly different (t(94) ≤ 1.62, *p* ≥ 0.11, d ≤ 0.37). Similarly, there were no significant differences between children in the high-risk-willingness group and the low-risk-willingness group for any of the perceived competences in active play items (t(94) ≤ 2.03, *p* ≥ 0.05, d ≤ 0.46).

## 4. Discussion

The current study had two aims: (1) to examine whether children with high risk willingness (HRW) displayed different motor behaviors compared to children with low risk willingness (LRW) when they freely explored and played in a virtual playground and (2) to examine if ratings of perceived gross motor competence were different between children with low risk willingness in the virtual playground compared to the children with high risk willingness. These aims were carried out by defining two groups of children based on their risk-taking behavior in the playground. One group was designated as low risk willingness (n = 29), consisting of children who did not jump onto any of the pillars and predominantly explored Zones 1–3 (see Figure 1), and the second group was designated as high-risk-willingness children (n = 89) who spent above-average time exploring Zone 4 and jumped at least one time on all three pillars.

The first aim was carried out by comparing whole-body movement variability and spatiotemporal parameters from the virtual playground. The findings indicated significantly greater overall exploration (higher mean speed and overall distance) and whole-body movement variability (see Table 2) in the high-risk-willingness group compared to the low-risk-willingness group of children, with relatively large effect sizes [62,63] for the between-group differences (d ≥ 0.8). As for the second aim, the two groups of children were compared on self-ratings of locomotor and active play items from the children’s Pictorial Scale of Perceived Movement Skill Competence [53,64]. The findings did not indicate any significant differences in the ratings of perceived motor competence (see Table 3) with small effect sizes (d ≤ 0.40) for the between-group differences. Thus, the children’s perceptions of their gross motor competence in the included play and locomotor items did not constitute a significant factor in terms of explaining differences in different risk willingness and motor behaviors in the playground.

The results from Research Question 1 showed that children with high risk willingness in the virtual playground explored a greater total distance (and thus moved at a higher average speed) and had higher mean accelerations compared to their low-risk-willingness counterparts (see Table 2). This result indicates more variations in speed for the HRW group when exploring the playground. It should be noted that a part of this variance comes from jumping to and from pillars, although these do not account for all changes in acceleration occurring during the three-minute period (i.e., the mean number of visits to pillars was six). Thus, exploring risk in the playground by jumping onto pillars follows alongside moving with both higher and greater variation in speed. Viewed through the lens of the model of children’s dynamic risk-taking [14], these results suggest that risk assessments during play lead to decisions that explore risk (i.e., jumping onto pillars and moving in Zone 4) and are integrated with risk-taking consisting of more dynamic and variable movements.

Indeed, motion capture analysis of upper- and lower-body movements indicated higher movement variability in the high-risk willingness group compared to the low-risk-taking children in the playground (see Table 2). This whole-body movement variability emerges as greater variation in foot placement accompanied by more variation in lower-body joint rotations, as well as more active arm lifting and movement in the HRW group of children. These children who visited and explored areas with higher levels of risk, to a greater extent, had to release degrees of freedom in their motor system to successfully complete the activity (i.e., not falling off). For example, jumping on pillars demands greater effort from children as they must strategically manage degrees of freedom to control acceleration, execute the jump, and maintain balance [65], all while navigating the inherent element of risk.

It has been postulated that, based on the theory of degrees of freedom [25], freezing the degrees of freedom is a movement strategy that indicates the simplification of control and displays less variability in the relationship between movements, the potential goal of the task, and the intentions of the children. By contrast, freeing degrees of freedom signifies better motor range, as children explore a higher number of degrees of freedom combinations and variability in task achievement [12,20,21,66]. This framework can explain the findings of the current study, as a better motor repertoire with more utilization of degrees of freedom in the high-risk-willingness group might have contributed to handling the risks as they moved fast in Zone 4 or jumped onto pillars in the virtual playground. A more adaptable and varied motor repertoire could thus relate to both handling specific task requirements (i.e., jumping) as well as the overall locomotor and balance features expressed in the exploratory behavior in the playground. These findings thus suggest that willingness to take and manage risks is inherently linked to and incorporates children’s bodily movements, although decisions to engage in risk-taking are considered primarily a cognitive process [67]. This seems to be aligned with ecological perspectives addressing that task-specific play behavior displayed by children emerges because of interacting and defining motor, perceptual, cognitive, and affective features [31,68].

The findings from Research Question 2 did not reveal any significant differences in perceived gross motor competence between the children displaying high risk willingness in the virtual playground compared to those expressing low risk willingness (see Table 3). Although high perceived motor competence has been found to positively affect children’s beliefs in themselves [69], the perceived gross motor competence rated by the children in the current study did not relate to their risk willingness in the playground.

There are methodological considerations that pertain to these results: First, Norwegian children in the seven-to-ten-year-old age group generally relate their competence as high or very high on the items included in the current study, contributing to potentially too little statistical variance for making comparisons. Second, children might not always have a realistic understanding of their gross motor competence or simply be unsure about their actual gross motor abilities, as both overestimation and underestimation have been reported previously [45,69]. Third, perceived motor competence captured by the PMSC is based on a self-evaluation of individual performance levels in specific motor tasks, and it is well-known that the assessment of actual motor performance is particularly prone to substantial inter- and intra-individual variability that, among other things, can be displayed as low correlations between performance for different motor tasks [70,71]. This specificity of performance in specific motor tasks might thus contribute to inconsistency in the self-evaluation of competence levels [52].

Apart from the methodological considerations, the null finding of the current study, regarding no differences in perceived motor competence between HRW and LRW children in the playground, needs to be interpreted in light of the scarcity of previous empirical examinations of the relationship between perceived motor competence and children’s actual play behavior. Indeed, little evidence is available for the relationship between play behavior and children’s actual gross motor development [45,64,70]. The specifics of the relationship between children’s perceived motor competence and their play behavior, including risk willingness, are thus an important consideration for further examination.

There are overall methodological limitations in the current study that warrant further examination. First, there was a smaller proportion of children designated as having low risk willingness compared to children categorized as high risk willingness. Firstly, this can be explained by the cultural context of Norway and children’s upbringing: Norwegian parents generally support their children’s exploration of nature and risky play, especially when having had similar experiences [72]. Similarly, kindergartens and schools can provide opportunities for risky play, and school practitioners are required to provide opportunities for children to engage in risky play, allowing them to gain experience and improve their risk-handling abilities [5,73]. Secondly, risk willingness is challenging to define clearly and even more difficult to investigate, as risk perceptions vary between individuals. This study employed elements associated with risky play as predictors to distinguish children who engaged in greater risk behaviors in the playground from those who exhibited fewer risk behaviors. Since the novel virtual reality playground only includes risky play in height, tasks incorporating other categories of risky play are needed to gain a broader understanding of children’s movement variability in exploratory and risky play.

## 5. Conclusions

The current study examined the relationship between children’s risk willingness and motor behavior in a virtual playground and the association with perceived gross motor competence. The findings indicate that children with high risk willingness demonstrated greater movement variability and engaged more actively with riskier areas of the playground than those with low risk willingness. These results suggest that risk-taking behavior is linked to a more flexible and adaptable motor repertoire, supporting the notion that children who embrace physical challenges exhibit greater variability in their movement strategies. However, no significant differences were found between the HRW and LRW groups in self-reported perceived gross motor competence. This suggests that risk willingness may be more closely associated with actual motor behavior than with perceived competence in the context of risky play in a playground. The lack of association between perceived gross motor competence and risk-taking behavior highlights the complexity of self-assessment in motor skills and calls for further research on how children perceive their own motor abilities. Overall, the study underscores the role of risk willingness in shaping children’s motor development and exploration strategies. Future research should further investigate the interaction between perceived and actual competence in play, as well as the long-term implications of risk-taking in motor learning and development across various play environments.

## Figures and Tables

**Table 1:** Demographics of the children with low risk willingness (LRW) and high risk willingness (HRW) in the virtual playground.

Variable		LRW (n = 29)	HRW (n = 67)
Girls/boys (n)	19/10	19/10	32/35
School	I	13	25
	II	10	21
	III	2	14
	IV	4	7
Grade	2	9	7
	3	17	25
	4	3	35
Tried VR before (no/yes)		15/13	22/40
If VR felt	Not realistic	1	3
	A bit realistic	3	6
	Quite realistic	12	35
	Very realistic	12	18
Virtual reality sickness (no/yes)		25/3	59/3
Want to try VR again (no/yes)		2/26	1/61

**Table 2:** Descriptive statistics of motor behavior variables across children with different risk willingness in virtual reality playground.

Variable	Title 2	LRW (n = 29)	HRW (n = 67)
Spatiotemporal Measures Fall (no/yes) Pillar visits (n) Time in zone (%) Distance (m) Velocity (m/s^2^) Acceleration (m/s^3^)		21/8	50/17
		0	5.9 (2.3)
	I II III IV	17.6 (10.4) 38.5 (12.6) 31.1 (12.2) 12.8 (0.07) 29.7 (8.5) 0.27 (0.08) 0.41 (0.09)	8.1 (0.05) 18.4 (0.05) 33.1 (0.06) 40.4 (0.06) 45.1 (12.5) 0.38 (0.09) 0.58 (0.12)
Whole-Body Movement Variability Head rotation (°) Shoulder abduction–adduction (°) Elbow flexion–extension (°) Arm lift (%) Hip flexion–extension (°) Knee flexion–extension (°) Ankle dorsiflexion–extension (°) Foot distance (m)	Pitch Yaw Mean Variability Anteroposterior Mediolateral Vertical	10.45 (7.19) 10.68 (5.03) 6.21 (2.96) 17.89 (12.66) 82.51 (0.04) 0.03 (0.01) 10.12 (1.93) 12.47 (2.33) 6.95 (1.21) 0.09 (0.02) 0.09 (0.02) 0.03 (0.01)	12.07 (8.31) 12.51 (5.49) 9.56 (3.11) 21.97 (9.68) 85.52 (0.04) 0.06 (0.03) 14.97 (3.72) 15.62 (3.17) 10.25 (1.72) 0.13 (0.02) 0.13 (0.02) 0.04 (0.01)

**Table 3:** Descriptive statistics (mean and SD) of perceived motor competence across children with different risk willingness in virtual reality playground.

Variable	LRW (n = 29)	HRW (n = 67)
Running	3.18 (0.77)	3.56 (0.56)
Galloping	2.25 (0.84)	2.50 (0.94)
Hopping	3.14 (0.71)	3.15 (0.67)
Skipping	2.57 (0.96)	2.84 (0.93)
Jumping	2.86 (0.89)	3.03 (0.73)
Sliding	3.11 (0.96)	2.93 (0.85)
Locomotion	2.85 (0.44)	3.01 (0.45)
Cycling	2.93 (0.85)	3.61 (0.64)
Scootering	2.89 (0.96)	3.31 (0.79)
Board Paddle	2.68 (1.09)	3.10 (0.88)
Skating	2.54 (1.17)	2.94 (1.17)
Swimming	2.25 (1.08)	2.98 (1.08)
Climbing	2.29 (1.08)	3.12 (0.78)
Active Play	2.79 (0.63)	2.98 (0.45)

## Data Availability

The data that support the findings of this study are available from the corresponding author (L.S.) upon reasonable request.
